# Does playing violent video games cause aggression? A longitudinal intervention study

**DOI:** 10.1038/s41380-018-0031-7

**Published:** 2018-03-13

**Authors:** Simone Kühn, Dimitrij Tycho Kugler, Katharina Schmalen, Markus Weichenberger, Charlotte Witt, Jürgen Gallinat

**Affiliations:** 1 Max Planck Institute for Human Development, Center for Lifespan Psychology, Lentzeallee 94, 14195 Berlin, Germany; 2 Clinic and Policlinic for Psychiatry and Psychotherapy, University Clinic Hamburg-Eppendorf, Martinistraße 52, 20246 Hamburg, Germany

**Keywords:** Psychology, Neuroscience

## Abstract

It is a widespread concern that violent video games promote aggression, reduce pro-social behaviour, increase impulsivity and interfere with cognition as well as mood in its players. Previous experimental studies have focussed on short-term effects of violent video gameplay on aggression, yet there are reasons to believe that these effects are mostly the result of priming. In contrast, the present study is the first to investigate the effects of long-term violent video gameplay using a large battery of tests spanning questionnaires, behavioural measures of aggression, sexist attitudes, empathy and interpersonal competencies, impulsivity-related constructs (such as sensation seeking, boredom proneness, risk taking, delay discounting), mental health (depressivity, anxiety) as well as executive control functions, before and after 2 months of gameplay. Our participants played the violent video game Grand Theft Auto V, the non-violent video game The Sims 3 or no game at all for 2 months on a daily basis. No significant changes were observed, neither when comparing the group playing a violent video game to a group playing a non-violent game, nor to a passive control group. Also, no effects were observed between baseline and posttest directly after the intervention, nor between baseline and a follow-up assessment 2 months after the intervention period had ended. The present results thus provide strong evidence against the frequently debated negative effects of playing violent video games in adults and will therefore help to communicate a more realistic scientific perspective on the effects of violent video gaming.

The concern that violent video games may promote aggression or reduce empathy in its players is pervasive and given the popularity of these games their psychological impact is an urgent issue for society at large. Contrary to the custom, this topic has also been passionately debated in the scientific literature. One research camp has strongly argued that violent video games increase aggression in its players [1, 2], whereas the other camp [3, 4] repeatedly concluded that the effects are minimal at best, if not absent. Importantly, it appears that these fundamental inconsistencies cannot be attributed to differences in research methodology since even meta-analyses, with the goal to integrate the results of all prior studies on the topic of aggression caused by video games led to disparate conclusions [2, 3]. These meta-analyses had a strong focus on children, and one of them [2] reported a marginal age effect suggesting that children might be even more susceptible to violent video game effects.

To unravel this topic of research, we designed a randomised controlled trial on adults to draw causal conclusions on the influence of video games on aggression. At present, almost all experimental studies targeting the effects of violent video games on aggression and/or empathy focussed on the effects of short-term video gameplay. In these studies the duration for which participants were instructed to play the games ranged from 4 min to maximally 2 h (mean = 22 min, median = 15 min, when considering all experimental studies reviewed in two of the recent major meta-analyses in the field [3, 5]) and most frequently the effects of video gaming have been tested directly after gameplay.

It has been suggested that the effects of studies focussing on consequences of short-term video gameplay (mostly conducted on college student populations) are mainly the result of priming effects, meaning that exposure to violent content increases the accessibility of aggressive thoughts and affect when participants are in the immediate situation [6]. However, above and beyond this the General Aggression Model (GAM, [7]) assumes that repeatedly primed thoughts and feelings influence the perception of ongoing events and therewith elicits aggressive behaviour as a long-term effect. We think that priming effects are interesting and worthwhile exploring, but in contrast to the notion of the GAM our reading of the literature is that priming effects are short-lived (suggested to only last for <5 min and may potentially reverse after that time [8]). Priming effects should therefore only play a role in very close temporal proximity to gameplay. Moreover, there are a multitude of studies on college students that have failed to replicate priming effects [9–11] and associated predictions of the so-called GAM such as a desensitisation against violent content [12–14] in adolescents and college students or a decrease of empathy [15] and pro-social behaviour [16, 17] as a result of playing violent video games.

However, in our view the question that society is actually interested in is not: “Are people more aggressive after having played violent video games for a few minutes? And are these people more aggressive minutes after gameplay ended?”, but rather “What are the effects of frequent, habitual violent video game playing? And for how long do these effects persist (not in the range of minutes but rather weeks and months)?” For this reason studies are needed in which participants are trained over longer periods of time, tested after a longer delay after acute playing and tested with broader batteries assessing aggression but also other relevant domains such as empathy as well as mood and cognition. Moreover, long-term follow-up assessments are needed to demonstrate long-term effects of frequent violent video gameplay. To fill this gap, we set out to expose adult participants to two different types of video games for a period of 2 months and investigate changes in measures of various constructs of interest at least one day after the last gaming session and test them once more 2 months after the end of the gameplay intervention. In contrast to the GAM, we hypothesised no increases of aggression or decreases in pro-social behaviour even after long-term exposure to a violent video game due to our reasoning that priming effects of violent video games are short-lived and should therefore not influence measures of aggression if they are not measured directly after acute gaming. In the present study, we assessed potential changes in the following domains: behavioural as well as questionnaire measures of aggression, empathy and interpersonal competencies, impulsivity-related constructs (such as sensation seeking, boredom proneness, risk taking, delay discounting), and depressivity and anxiety as well as executive control functions. As the effects on aggression and pro-social behaviour were the core targets of the present study, we implemented multiple tests for these domains. This broad range of domains with its wide coverage and the longitudinal nature of the study design enabled us to draw more general conclusions regarding the causal effects of violent video games.

## Materials and methods

### Participants

Ninety healthy participants (mean age = 28 years, SD = 7.3, range: 18–45, 48 females) were recruited by means of flyers and internet advertisements. The sample consisted of college students as well as of participants from the general community. The advertisement mentioned that we were recruiting for a longitudinal study on video gaming, but did not mention that we would offer an intervention or that we were expecting training effects. Participants were randomly assigned to the three groups ruling out self-selection effects. The sample size was based on estimates from a previous study with a similar design [18]. After complete description of the study, the participants’ informed written consent was obtained. The local ethics committee of the Charité University Clinic, Germany, approved of the study. We included participants that reported little, preferably no video game usage in the past 6 months (none of the participants ever played the game *Grand Theft Auto V (GTA)* or *Sims 3* in any of its versions before). We excluded participants with psychological or neurological problems. The participants received financial compensation for the testing sessions (200 Euros) and performance-dependent additional payment for two behavioural tasks detailed below, but received no money for the training itself.

### Training procedure

The violent video game group (5 participants dropped out between pre- and posttest, resulting in a group of *n* = 25, mean age = 26.6 years, SD = 6.0, 14 females) played the game *Grand Theft Auto V* on a Playstation 3 console over a period of 8 weeks. The active control group played the non-violent video game *Sims 3* on the same console (6 participants dropped out, resulting in a group of *n* = 24, mean age = 25.8 years, SD = 6.8, 12 females). The passive control group (2 participants dropped out, resulting in a group of *n* = 28, mean age = 30.9 years, SD = 8.4, 12 females) was not given a gaming console and had no task but underwent the same testing procedure as the two other groups. The passive control group was not aware of the fact that they were part of a control group to prevent self-training attempts. The experimenters testing the participants were blind to group membership, but we were unable to prevent participants from talking about the game during testing, which in some cases lead to an unblinding of experimental condition. Both training groups were instructed to play the game for at least 30 min a day. Participants were only reimbursed for the sessions in which they came to the lab. Our previous research suggests that the perceived fun in gaming was positively associated with training outcome [18] and we speculated that enforcing training sessions through payment would impair motivation and thus diminish the potential effect of the intervention. Participants underwent a testing session before (baseline) and after the training period of 2 months (posttest 1) as well as a follow-up testing sessions 2 months after the training period (posttest 2).

### Grand Theft Auto V (GTA)

GTA is an action-adventure video game situated in a fictional highly violent game world in which players are rewarded for their use of violence as a means to advance in the game. The single-player story follows three criminals and their efforts to commit heists while under pressure from a government agency. The gameplay focuses on an open world (sandbox game) where the player can choose between different behaviours. The game also allows the player to engage in various side activities, such as action-adventure, driving, third-person shooting, occasional role-playing, stealth and racing elements. The open world design lets players freely roam around the fictional world so that gamers could in principle decide not to commit violent acts.

### The Sims 3 (Sims)

Sims is a life simulation game and also classified as a sandbox game because it lacks clearly defined goals. The player creates virtual individuals called “Sims”, and customises their appearance, their personalities and places them in a home, directs their moods, satisfies their desires and accompanies them in their daily activities and by becoming part of a social network. It offers opportunities, which the player may choose to pursue or to refuse, similar as GTA but is generally considered as a pro-social and clearly non-violent game.

### Assessment battery

To assess aggression and associated constructs we used the following questionnaires: Buss–Perry Aggression Questionnaire [19], State Hostility Scale [20], Updated Illinois Rape Myth Acceptance Scale [21, 22], Moral Disengagement Scale [23, 24], the Rosenzweig Picture Frustration Test [25, 26] and a so-called World View Measure [27]. All of these measures have previously been used in research investigating the effects of violent video gameplay, however, the first two most prominently. Additionally, behavioural measures of aggression were used: a Word Completion Task, a Lexical Decision Task [28] and the Delay frustration task [29] (an inter-correlation matrix is depicted in Supplementary Figure 11). From these behavioural measures, the first two were previously used in research on the effects of violent video gameplay. To assess variables that have been related to the construct of impulsivity, we used the Brief Sensation Seeking Scale [30] and the Boredom Propensity Scale [31] as well as tasks assessing risk taking and delay discounting behaviourally, namely the Balloon Analogue Risk Task [32] and a Delay-Discounting Task [33]. To quantify pro-social behaviour, we employed: Interpersonal Reactivity Index [34] (frequently used in research on the effects of violent video gameplay), Balanced Emotional Empathy Scale [35], Reading the Mind in the Eyes test [36], Interpersonal Competence Questionnaire [37] and Richardson Conflict Response Questionnaire [38]. To assess depressivity and anxiety, which has previously been associated with intense video game playing [39], we used Beck Depression Inventory [40] and State Trait Anxiety Inventory [41]. To characterise executive control function, we used a Stop Signal Task [42], a Multi-Source Interference Task [43] and a Task Switching Task [44] which have all been previously used to assess effects of video gameplay. More details on all instruments used can be found in the Supplementary Material.

### Data analysis

On the basis of the research question whether violent video game playing enhances aggression and reduces empathy, the focus of the present analysis was on time by group interactions. We conducted these interaction analyses separately, comparing the violent video game group against the active control group (GTA vs. Sims) and separately against the passive control group (GTA vs. Controls) that did not receive any intervention and separately for the potential changes during the intervention period (baseline vs. posttest 1) and to test for potential long-term changes (baseline vs. posttest 2). We employed classical frequentist statistics running a repeated-measures ANOVA controlling for the covariates sex and age.

Since we collected 52 separate outcome variables and conduced four different tests with each (GTA vs. Sims, GTA vs. Controls, crossed with baseline vs. posttest 1, baseline vs. posttest 2), we had to conduct 52 × 4 = 208 frequentist statistical tests. Setting the alpha value to 0.05 means that by pure chance about 10.4 analyses should become significant. To account for this multiple testing problem and the associated alpha inflation, we conducted a Bonferroni correction. According to Bonferroni, the critical value for the entire set of *n* tests is set to an alpha value of 0.05 by taking alpha/*n* = 0.00024.

Since the Bonferroni correction has sometimes been criticised as overly conservative, we conducted false discovery rate (FDR) correction [45]. FDR correction also determines adjusted *p*-values for each test, however, it controls only for the number of false discoveries in those tests that result in a discovery (namely a significant result).

Moreover, we tested for group differences at the baseline assessment using independent *t*-tests, since those may hamper the interpretation of significant interactions between group and time that we were primarily interested in.

Since the frequentist framework does not enable to evaluate whether the observed null effect of the hypothesised interaction is indicative of the absence of a relation between violent video gaming and our dependent variables, the amount of evidence in favour of the null hypothesis has been tested using a Bayesian framework. Within the Bayesian framework both the evidence in favour of the null and the alternative hypothesis are directly computed based on the observed data, giving rise to the possibility of comparing the two. We conducted Bayesian repeated-measures ANOVAs comparing the model in favour of the null and the model in favour of the alternative hypothesis resulting in a Bayes factor (BF) using Bayesian Information criteria [46]. The BF_01_ suggests how much more likely the data is to occur under the null hypothesis. All analyses were performed using the JASP software package (https://jasp-stats.org).

## Results

Sex distribution in the present study did not differ across the groups (*χ*^2^ *p*-value > 0.414). However, due to the fact that differences between males and females have been observed in terms of aggression and empathy [47], we present analyses controlling for sex. Since our random assignment to the three groups did result in significant age differences between groups, with the passive control group being significantly older than the GTA (*t*(51) = −2.10, *p* = 0.041) and the Sims group (*t*(50) = −2.38, *p* = 0.021), we also controlled for age.

The participants in the violent video game group played on average 35 h and the non-violent video game group 32 h spread out across the 8 weeks interval (with no significant group difference *p* = 0.48).

To test whether participants assigned to the violent GTA game show emotional, cognitive and behavioural changes, we present the results of repeated-measure ANOVA time x group interaction analyses separately for GTA vs. Sims and GTA vs. Controls (Tables 1–3). Moreover, we split the analyses according to the time domain into effects from baseline assessment to posttest 1 (Table 2) and effects from baseline assessment to posttest 2 (Table 3) to capture more long-lasting or evolving effects. In addition to the statistical test values, we report partial omega squared (*ω*^2^) as an effect size measure. Next to the classical frequentist statistics, we report the results of a Bayesian statistical approach, namely BF_01_, the likelihood with which the data is to occur under the null hypothesis that there is no significant time × group interaction. In Table 2, we report the presence of significant group differences at baseline in the right most column.

**Table 1:** Mean and standard deviation (in brackets) split according to group and time point for the dependent variables

Dependent variables	GTA			Sims			Controls		
	Baseline	Posttest 1	Posttest 2	Baseline	Posttest 1	Posttest 2	Baseline	Posttest 1	Posttest 2
Questionnaires assessing aggression and associated constructs									
BP physical aggression	1.99 (0.76)	2.03 (0.89)	1.95 (0.82)	1.55 (0.47)	1.66 (0.62)	1.50 (0.38)	1.94 (0.92)	1.87 (0.93)	2.08 (1.26)
BP verbal aggression	3.78 (0.80)	3.61 (0.92)	3.61 (0.77)	3.62 (1.04)	3.63 (0.83)	3.56 (0.69)	3.66 (1.18)	3.82 (1.14)	3.86 (1.15)
BP anger	2.85 (1.08)	2.63 (0.98)	2.61 (1.21)	2.42 (0.77)	2.39 (0.92)	2.22 (0.77)	2.64 (1.34)	2.49 (1.16)	2.72 (1.32)
BP hostility	1.89 (0.66)	2.01 (0.98)	2.13 (0.83)	1.76 (0.68)	1.89 (0.74)	1.73 (0.69)	2.09 (1.20)	2.11 (1.23)	2.37 (1.32)
SHS feeling mean	1.17 (0,22)	1.27 (0.51)	1.17 (0.20)	1.15 (0.28)	1.17 (0.26)	1.26 (0.52)	1.36 (0.59)	1.33 (0.56)	1.32 (0.48)
SHS aggravation	1.34 (0.48)	1.41 (0.61)	1.29 (0.37)	1.32 (0.45)	1.32 (0.48)	1.37 (0.63)	1.46 (0.66)	1.48 (0.68)	1.45 (0.62)
SHS feeling unsociable	1.37 (0.60)	1.27 (0.29)	1.24 (0.38)	1.28 (0.51)	1.17 (0.26)	1.30 (0.61)	1.49 (0.66)	1.45 (0.55)	1.36 (0.54)
SHS lack of positive feelings	2.10 (0.68)	2.06 (0.58)	1.99 (0.61)	2.00 (0.62)	1.93 (0.48)	2.03 (0.59)	2.20 (0.82)	2.18 (0.80)	2.28 (0.93)
IRMAS she asked for it	10.80 (3.37)	10.68 (3.81)	10.39 (4.75)	11.08 (3.43)	9.92 (2.50)	10.10 (2.97)	9.75 (4.12)	10.18 (3.77)	9.74 (4.21)
IRMAS he did not mean to	12.16 (4.27)	13.28 (7.40)	10.39 (4.19)	11.29 (3.46)	10.46 (3.15)	9.67 (2.90)	10.68 (4.18)	9.75 (4.13)	8.91 (3.90)
IRMAS it was not really rape	6.84 (2.66)	6.68 (2.73)	6.00 (1.33)	6.38 (2.41)	6.17 (1.79)	5.67 (1.39)	7.11 (3.33)	7.07 (3.38)	6.61 (3.14)
IRMAS she lied	9.48 (3.61)	10.24 (3.31)	9.22 (3.32)	9.54 (3.68)	8.42 (3.31)	8.57 (3.79)	9.86 (4.93)	9.61 (4.93)	9.65 (4.99)
Moral Disengagement Scale	1.85 (0.54)	1.88 (0.58)	1.81 (0.65)	1.67 (0.40)	1.77 (0.59)	1.62 (0.43)	1.69 (0.56)	1.64 (0.56)	1.70 (0.62)
RPFT extra-punitive	0.46 (0.11)	0.46 (0.09)	0.45 (0.16)	0.46 (0.15)	0.46 (0.12)	0.47 (0.14)	0.53 (0.11)	0.49 (0.11)	0.49 (0.16)
RPFT intra-punitive	0.25 (0.09)	0.27 (0.08)	0.24 (0.07)	0.26 (0.09)	0.27 (0.08)	0.23 (0.05)	0.23 (0.06)	0.26 (0.06)	0.24 (0.06)
RPFT non-punitive	0.28 (0.08)	0.29 (0.10)	0.24 (0.12)	0.26 (0.08)	0.26 (0.08)	0.28 (0.10)	0.25 (0.09)	0.25 (0.08)	0.27 (0.12)
RPFT obstacle dominance	0.27 (0.09)	0.27 (0.10)	0.24 (0.12)	0.32 (0.07)	0.35 (0.08)	0.30 (0.09)	0.29 (0.10)	0.30 (0.08)	0.23 (0.08)
RPFT ego-defense	0.55 (0.09)	0.48 (0.11)	0.47 (0.16)	0.44 (0.08)	0.46 (0.06)	0.47 (0.10)	0.47 (0.09)	0.42 (0.11)	0.46 (0.12)
RPFT need persistence	0.18 (0.11)	0.25 (0.13)	0.23 (0.13)	0.23 (0.12)	0.20 (0.11)	0.23 (0.12)	0.23 (0.11)	0.26 (0.13)	0.28 (0.11)
WVM percent	26.43 (22.93)	19.37 (16.34)	18.90 (16.76)	18.73 (20.85)	12.35 (12.67)	11.91 (14.73)	15.16 (16.17)	16.39 (21.83)	18.61 (26.13)
WVM safety	9.28 (3.21)	9.28 (3.46)	9.33 (3.48)	10.83 (2.55)	11.29 (2.14)	10.67 (2.24)	11.00 (2.69)	10.96 (2.50)	10.61 (2.08)
Behavioral measures assessing aggression									
Word completion test	0.11 (0.06)	0.19 (0.06)	0.19 (0.05)	0.15 (0.05)	0.17 (0.07)	0.16 (0.05)	0.12 (0.05)	0.19 (0.11)	0.18 (0.07)
Lexical decision task	2.17 (2.30)	1.92 (1.80)	1.56 (1.30)	4.58 (8.70)	3.85 (7.81)	1.16 (1.46)	1.77 (1.97)	2.76 (7.03)	1.64 (2.13)
Delay frustration task	2.35 (2.31)	1.79 (1.69)	1.41 (1.23)	5.21 (9.16)	3.68 (7.73)	1.28 (1.58)	1.76 (1.96)	2.72 (6.90)	1.62 (2.14)
Sensation seeking and boredom proneness									
BSSS experience seeking	4.3 (0.92)	4.28 (0.94)	4.36 (0.94)	4.33 (0.76)	4.50 (0.72)	4.38 (0.84)	4.11 (0.70)	4.21 (0.78)	4.24 (0.80)
BSSS boredom susceptibility	3.34 (0.89)	3.24 (0.98)	3.06 (1.01)	3.42 (0.58)	3.42 (0.67)	3.26 (0.70)	2.93 (0.73)	3.16 (0.73)	3.20 (0.79)
BSSS thrill and adventure seeking	2.36 (1.01)	2.22 (1.15)	2.53 (1.17)	2.35 (1.18)	2.46 (1.08)	2.60 (1.14)	2.02 (0.91)	2.20 (0.90)	2.22 (0.95)
BSSS disinhibition	2.78 (1.09)	2.96 (1.08)	2.92 (1.28)	2.90 (1.15)	3.06 (1.19)	2.95 (1.17)	2.80 (0.81)	3.02 (0.93)	2.89 (0.95)
Boredom proneness scale	81.04 (17.57)	82.28 (16.83)	81.39 (19.92)	78.83 (16.62)	81.67 (17.96)	80.29 (19.07)	82.39 (19.63)	80.71 (19.57)	86.43 (20.03)
Behavioral measures assessing risk taking and delay discounting									
Balloon analogue risk task	31.75 (13.75)	39.49 (10.86)	41.56 (12.21)	39.60 (14.08)	43.89 (13.81)	46.42 (11.42)	35.57 (13.56)	41.65 (14.08)	48.65 (13.18)
Delay-discounting task delay	5.22 (24.85)	0.03 (0.08)	25.34 (123.25)	0.05 (0.12)	0.03 (0.08)	0.76 (2.46)	0.17 (0.80)	0.03 (0.10)	0.03 (0.07)
Delay-discounting task probability	14.26 (35.50)	8.78 (23.47)	12.00 (33.39)	3.70 (8.86)	1.94 (2.03)	2.17 (3.22)	17.99 (42.92)	3.27 (7.51)	4.61 (9.79)
Questionnaires and tests assessing empathy and interpersonal competence									
IRI perspective taking	2.43 (0.80)	2.50 (0.66)	2.57 (0.64)	2.70 (0.49)	2.71 (0.57)	2.57 (0.54)	2.63 (0.65)	2.70 (0.53)	2.45 (0.68)
IRI fantasy scale	2.05 (0.78)	2.04 (1.00)	1.28 (1.09)	2.48 (0.71)	1.81 (1.15)	1.41 (1.17)	2.15 (0.81)	1.96 (0.99)	1.56 (1.15)
IRI empathic concern	2.58 (0.72)	2.52 (0.54)	2.52 (0.56)	2.64 (0.52)	2.66 (0.51)	2.61 (0.59)	2.60 (0.54)	2.50 (0.55)	2.51 (0.51)
IRI personal distress	1.57 (0.55)	1.45 (0.75)	1.03 (0.91)	1.63 (0.57)	1.31 (0.87)	1.13 (0.95)	1.58 (0.74)	1.44 (0.82)	1.15 (0.93)
Balanced Emotional Empathy scale	6.23 (0.83)	6.13 (0.84)	6.12 (0.86)	6.31 (0.90)	6.45 (0.96)	6.27 (0.83)	6.20 (0.95)	6.22 (0.88)	6.01 (0.90)
Reading the mind in the eyes	25.40 (3.97)	25.00 (4.14)	27.28 (4.23)	25.54 (4.43)	26.04 (4.19)	26.79 (3.8)	24.69 (4.20)	25.31 (4.43)	24.58 (5.37)
ICQ initiating relationships	3.47 (0.66)	3.35 (0.76)	3.85 (1.73)	3.36 (0.58)	3.28 (0.53)	3.34 (0.66)	3.31 (0.86)	3.27 (0.95)	3.30 (0.93)
ICQ negative assertion	3.39 (0.67)	3.11 (0.67)	3.04 (0.77)	3.13 (0.66)	3.09 (0.74)	3.09 (0.81)	3.21 (0.61)	3.22 (0.66)	3.00 (0.72)
ICQ disclosing personal information	3.33 (0.68)	3.30 (0.73)	3.29 (0.83)	3.54 (0.53)	3.54 (0.58)	3.60 (0.56)	3.09 (0.81)	3.15 (0.78)	3.10 (0.89)
ICQ providing emotional support	4.14 (0.86)	4.01 (0.75)	4.09 (0.63)	4.10 (0.57)	4.01 (0.65)	4.09 (0.49)	3.98 (0.63)	3.97 (0.56)	3.99 (0.67)
ICQ advice and managing social conflict	3.24 (0.73)	3.31 (0.73)	3.38 (0.74)	3.59 (0.42)	3.57 (0.46)	3.62 (0.47)	3.47 (0.50)	3.49 (0.65)	3.45 (0.63)
RCRQ direct aggression	1.27 (0.35)	1.26 (0.30)	1.22 (0.29)	1.18 (0.24)	1.20 (0.23)	1.18 (0.19)	1.29 (0.44)	1.33 (0.52)	1.28 (0.56)
RCRQ indirect aggression	1.30 (0.26)	1.32 (0.36)	1.39 (0.31)	1.34 (0.23)	1.35 (0.31)	1.41 (0.36)	1.55 (0.49)	1.51 (0.53)	1.48 (0.47)
Questionnaires assessing depressivity and anxiety									
Beck depression inventory	3.64 (3.49)	4.04 (6.03)	4.28 (4.87)	2.88 (2.94)	3.88 (5.13)	3.05 (5.90)	5.11 (6.26)	4.32 (7.18)	4.48 (6.54)
STAI state	34.36 (6.36)	34.12 (8.82)	31.78 (6.84)	33.88 (8.67)	33.54 (9.78)	32.33 (10.31)	36.39 (10.65)	34.64 (6.68)	35.67 (9.85)
STAI trait	38.84 (6.90)	38.28 (7.58)	36.39 (8.38)	37.75 (7.54)	38.63 (8.30)	37.90 (8.57)	39.93 (7.68)	40.18 (9.64)	39.08 (9.51)
Behavioral measures assessing executive control function									
Stop signal task	212.2 (51.37)	193.0 (21.20)	188.3 (24.78)	204.9 (31.42)	198.1 (39.53)	195.3 (28.88)	226.5 (40.74)	209.8 (37.30)	201.0 (39.54)
Multi-source interference task	314.2 (55.29)	286.2 (54.39)	267.8 (46.66)	297.7 (49.42)	261.6 (53.75)	249.5 (50.36)	320.0 (88.71)	287.9 (63.85)	258.0 (64.29)
TS switching costs	69.71 (39.29)	61.92 (43.16)	36.59 (43.94)	62.18 (36.61)	40.45 (35.11)	47.82 (46.67)	70.18 (42.79)	47.59 (53.21)	46.91 (34.18)
TS mixing costs	133.0 (41.03)	105.0 (61.42)	64.82 (53.88)	112.10 (68.27)	82.10 (69.12)	103.45 (61.55)	126.1 (72.26)	124.5 (74.83)	86.90 (50.85)

*BP* Buss–Perry Aggression Questionnaire, *SHS* State Hostility Scale, *IRMAS* Updated Illinois Rape Myth Acceptance Scale, *RPFT* Rosenzweig Picture Frustration Test, *WVM* World View Measure, *BSSS* Brief Sensation Seeking Scale, *IRI* Interpersonal Reactivity Index, *ICQ* Interpersonal Competence Questionnaire, *RCRQ* Richardson Conflict Response Questionnaire, *STAI* State Trait Anxiety Inventory, *TS* Task switching

**Table 2:** Results of the statistical analysis using a classical frequentist approach and a Bayesian approach. Reported are the results of the group × time interaction while focusing on effects between baseline assessment and posttest 1 and controlling for the confounding variables age and sex. The outer right columns report group differences at the baseline assessment.

Dependent variables	GTA vs. Sims		GTA vs. Controls		Baseline *t*-test differences	
	Classical frequentist approach	Bayesian approach	Classical frequentist approach	Bayesian approach	GTA vs. Sims	GTA vs. Control
Questionnaires assessing aggression and associated constructs						
BP physical aggression	*F*(1,45) = 0.319, *p* = 0.575, *ω*^2^ < 0.001	BF_01_ = 2.400	*F*(1,49) = 0.782, *p* = 0.381, *ω*^2^ < 0.001	BF_01_ = 2.777	*p* = 0.030	
BP verbal aggression	*F*(1,45) = 1.288, *p* = 0.262, *ω*^2^ = 0.006	BF_01_ = 2.124	*F*(1,49) = 1.609, *p* = 0.211, *ω*^2^ = 0.011	BF_01_ = 1.006		
BP anger	*F*(1,45) = 0.885, *p* = 0.352, *ω*^2^ < 0.001	BF_01_ = 2.421	*F*(1,49) = 0.005, *p* = 0.943, *ω*^2^ < 0.001	BF_01_ = 3.428		
BP hostility	*F*(1,45) = 0.004, *p* = 0.947, *ω*^2^ < 0.001	BF_01_ = 3.259	*F*(1,49) = 0.118, *p* = 0.733, *ω*^2^ < 0.001	BF_01_ = 3.402		
SHS feeling mean	*F*(1,45) = 0.764, *p* = 0.387, *ω*^2^ < 0.001	BF_01_ = 2.603	*F*(1,49) = 0.808, *p* = 0.373, *ω*^2^ < 0.001	BF_01_ = 2.147		
SHS aggravation	*F*(1,45) = 0.370, *p* = 0.546, *ω*^2^ < 0.001	BF_01_ = 3.392	*F*(1,49) = 0.045, *p* = 0.834, *ω*^2^ < 0.001	BF_01_ = 3.893		
SHS feeling unsociable	*F*(1,45) = 0.000, *p* = 0.987, *ω*^2^ < 0.001	BF_01_ = 3.283	*F*(1,49) = 0.045, *p* = 0.833, *ω*^2^ < 0.001	BF_01_ = 2.985		
SHS lack of positive feelings	*F*(1,45) = 0.049, *p* = 0.825, *ω*^2^ < 0.001	BF_01_ = 3.597	*F*(1,49) = 0.005, *p* = 0.942, *ω*^2^ < 0.001	BF_01_ = 3.734		
IRMAS she asked for it	*F*(1,45) = 1.888, *p* = 0.176, *ω*^2^ = 0.018	BF_01_ = 1.669	*F*(1,49) = 0.457, *p* = 0.502, *ω*^2^ < 0.001	BF_01_ = 3.183		
IRMAS he did not mean to	*F*(1,45) = 1.763, *p* = 0.191, *ω*^2^ = 0.015	BF_01_ = 1.919	*F*(1,49) = 0.823, *p* = 0.369, *ω*^2^ < 0.001	BF_01_ = 1.520		
IRMAS it was not really rape	*F*(1,45) = 0.011, *p* = 0.915, *ω*^2^ < 0.001	BF_01_ = 3.583	*F*(1,49) = 0.301, *p* = 0.586, *ω*^2^ < 0.001	BF_01_ = 3.774		
IRMAS she lied	*F*(1,45) = 4.634, *p* = 0.037*, *ω*^2^ = 0.071	BF_01_ = 0.480	*F*(1,49) = 1.620, *p* = 0.209, *ω*^2^ = 0.011	BF_01_ = 1.722		
Moral Disengagement Scale	*F*(1,43) = 0.333, *p* = 0.567, *ω*^2^ < 0.001	BF_01_ = 2.815	*F*(1,45) = 0.919, *p* = 0.343, *ω*^2^ < 0.001	BF_01_ = 2.173		
RPFT extra-punitive	*F*(1,40) = 0.588, *p* = 0.448, *ω*^2^ < 0.001	BF_01_ = 2.725	*F*(1,40) = 0.287, *p* = 0.595, *ω*^2^ < 0.001	BF_01_ = 2.125		*p* = 0.028
RPFT intra-punitive	*F*(1,38) = 0.212, *p* = 0.648, *ω*^2^ < 0.001	BF_01_ = 2.957	*F*(1,40) = 0.004, *p* = 0.952, *ω*^2^ < 0.001	BF_01_ = 3.149		
RPFT non-punitive	*F*(1,39) = 0.001, *p* = 0.981, *ω*^2^ < 0.001	BF_01_ = 2.989	*F*(1,41) = 0.019, *p* = 0.891, *ω*^2^ < 0.001	BF_01_ = 3.697		
RPFT obstacle dominance	*F*(1,39) = 2.564, *p* = 0.117, *ω*^2^ = 0.035	BF_01_ = 1.944	*F*(1,40) = 0.186, *p* = 0.669, *ω*^2^ < 0.001	BF_01_ = 2.903		
RPFT ego-defense	*F*(1,40) = 0.127, *p* = 0.723, *ω*^2^ < 0.001	BF_01_ = 0.142	*F*(1,41) = 1.790, *p* = 0.188, *ω*^2^ = 0.016	BF_01_ = 2.701	*p* < 0.001	*p* = 0.004
RPFT need persistence	*F*(1,40) = 1.406, *p* = 0.243, *ω*^2^ = 0.007	BF_01_ = 0.104	*F*(1,41) = 3.310, *p* = 0.076, *ω*^2^ = 0.041	BF_01_ = 2.366		
WVM percent	*F*(1,44) = 0.041, *p* = 0.840, *ω*^2^ < 0.001	BF_01_ = 3.395	*F*(1,49) = 5.914, *p* = 0.019*, *ω*^2^ = 0.079	BF_01_ = 0.741		*p* = 0.025
WVM safety	*F*(1,45) = 0.894, *p* = 0.349, *ω*^2^ < 0.001	BF_01_ = 3.112	*F*(1,48) = 0.004, *p* = 0.995, *ω*^2^ < 0.001	BF_01_ = 3.579		*p* = 0.045
Behavioral measures assessing aggression						
Word completion test	*F*(1,42) = 5.958, *p* = 0.019*, *ω*^2^ = 0.084	BF_01_ = 0.298	*F*(1,41) = 0.001, *p* = 0.872, *ω*^2^ < 0.001	BF_01_ = 3.282	*p* = 0.013	
Lexical decision task	*F*(1,47) = 0.878, *p* = 0.353, *ω*^2^ < 0.001	BF_01_ = 2.854	*F*(1,49) = 0.126, *p* = 0.724, *ω*^2^ < 0.001	BF_01_ = 3.089		
Delay frustration task	*F*(1,43) = 0.087, *p* = 0.770, *ω*^2^ < 0.001	BF_01_ = 3.853	*F*(1,43) = 1.141, *p* = 0.291, *ω*^2^ = 0.002	BF_01_ = 2.722		
Sensation seeking and boredom proneness						
BSSS experience seeking	*F*(1,45) = 1.796, *p* = 0.187, *ω*^2^ = 0.015	BF_01_ = 1.323	*F*(1,49) = 2.053, *p* = 0.158, *ω*^2^ = 0.015	BF_01_ = 2.169		
BSSS boredom susceptibility	*F*(1,45) = 0.200, *p* = 0.657, *ω*^2^ < 0.001	BF_01_ = 2.936	*F*(1,49) = 2.177, *p* = 0.146, *ω*^2^ = 0.022	BF_01_ = 0.915		
BSSS thrill and adventure seeking	*F*(1,45) = 0.905, *p* = 0.346, *ω*^2^ < 0.001	BF_01_ = 2.279	*F*(1,49) = 2.398, *p* = 0.128, *ω*^2^ = 0.026	BF_01_ = 1.109		
BSSS disinhibition	*F*(1,45) = 0.023, *p* = 0.879, *ω*^2^ < 0.001	BF_01_ = 3.408	*F*(1,49) = 0.009, *p* = 0.925, *ω*^2^ < 0.001	BF_01_ = 3.463		
Boredom proneness scale	*F*(1,45) = 0.490, *p* = 0.487, *ω*^2^ < 0.001	BF_01_ = 2.863	*F*(1,49) = 3.599, *p* = 0.064, *ω*^2^ = 0.042	BF_01_ = 2.172		
Behavioral measures assessing risk taking and delay discounting						
Balloon analogue risk task	*F*(1,47) = 0.893, *p* = 0.349, *ω*^2^ < 0.001	BF_01_ = 2.216	*F*(1,47) = 0.383, *p* = 0.539, *ω*^2^ < 0.001	BF_01_ = 3.267	*p* = 0.021	
Delay-discounting task delay	*F*(1,45) = 1.316, *p* = 0.257, *ω*^2^ = 0.006	BF_01_ = 2.841	*F*(1,47) = 1.304, *p* = 0.259, *ω*^2^ = 0.006	BF_01_ = 1.926		
Delay-discounting task probability	*F*(1,45) = 0.316, *p* = 0.577, *ω*^2^ < 0.001	BF_01_ = 3.036	*F*(1,47) = 0.427, *p* = 0.516, *ω*^2^ < 0.001	BF_01_ = 2.837		
Questionnaires and tests assessing empathy and interpersonal competence						
IRI perspective taking	*F*(1,46) = 0.061, *p* = 0.807, *ω*^2^ < 0.001	BF_01_ = 3.538	*F*(1,50) = 0.016, *p* = 0.899, *ω*^2^ < 0.001	BF_01_ = 3.817		
IRI fantasy scale	*F*(1,56) = 5.211, *p* = 0.026*, *ω*^2^ = 0.057	BF_01_ = 0.402	*F*(1,56) = 1.131, *p* = 0.292, *ω*^2^ = 0.002	BF_01_ = 3.140		
IRI empathic concern	*F*(1,46) = 0.365, *p* = 0.549, *ω*^2^ < 0.001	BF_01_ = 2.949	*F*(1,50) = 0.057, *p* = 0.813, *ω*^2^ < 0.001	BF_01_ = 3.480		
IRI personal distress	*F*(1,56) = 1.192, *p* = 0.280, *ω*^2^ = 0.003	BF_01_ = 2.484	*F*(1,56) = 0.051, *p* = 0.821, *ω*^2^ < 0.001	BF_01_ = 3.934		
Balanced emotional empathy scale	*F*(1,44) = 2.895, *p* = 0.096, *ω*^2^ = 0.038	BF_01_ = 1.264	*F*(1,48) = 0.517, *p* = 0.476, *ω*^2^ < 0.001	BF_01_ = 2.984		
Reading the mind in the eyes	*F*(1,45) = 0.915, *p* = 0.344, *ω*^2^ < 0.001	BF_01_ = 2.319	*F*(1,50) = 0.997, *p* = 0.323, *ω*^2^ < 0.001	BF_01_ = 2.403		
ICQ initiating relationships	*F*(1,45) = 0.039, *p* = 0.844, *ω*^2^ < 0.001	BF_01_ = 3.447	*F*(1,49) = 0.337, *p* = 0.564, *ω*^2^ < 0.001	BF_01_ = 3.134		
ICQ negative assertion	*F*(1,45) = 2.793, *p* = 0.102, *ω*^2^ = 0.037	BF_01_ = 0.973	*F*(1,49) = 2.551, *p* = 0.117, *ω*^2^ = 0.029	BF_01_ = 1.093		
ICQ disclosing personal information	*F*(1,45) = 0.081, *p* = 0.777, *ω*^2^ < 0.001	BF_01_ = 3.217	*F*(1,49) = 0.773, *p* = 0.384, *ω*^2^ < 0.001	BF_01_ = 2.966		
ICQ providing emotional support	*F*(1,45) = 0.027, *p* = 0.870, *ω*^2^ < 0.001	BF_01_ = 3.754	*F*(1,49) = 0.634, *p* = 0.430, *ω*^2^ < 0.001	BF_01_ = 3.775		
ICQ advice and managing social conflict	*F*(1,45) = 0.253, *p* = 0.617, *ω*^2^ < 0.001	BF_01_ = 3.077	*F*(1,49) = 0.194, *p* = 0.661, *ω*^2^ < 0.001	BF_01_ = 3.579		
RCRQ direct aggression	*F*(1,45) = 0.100, *p* = 0.753, *ω*^2^ < 0.001	BF_01_ = 3.160	*F*(1,49) = 0.074, *p* = 0.787, *ω*^2^ < 0.001	BF_01_ = 3.344		
RCRQ indirect aggression	*F*(1,45) = 0.010, *p* = 0.922, *ω*^2^ < 0.001	BF_01_ = 3.377	*F*(1,49) = 0.141, *p* = 0.709, *ω*^2^ < 0.001	BF_01_ = 3.378		*p* = 0.009
Questionnaires assessing depressivity and anxiety						
Beck depression inventory	*F*(1,45) = 0.398, *p* = 0.531, *ω*^2^ < 0.001	BF_01_ = 3.230	*F*(1,49) = 0.540, *p* = 0.466, *ω*^2^ < 0.001	BF_01_ = 2.278		
STAI state	*F*(1,45) = 0.009, *p* = 0.923, *ω*^2^ < 0.001	BF_01_ = 3.185	*F*(1,49) = 0.022, *p* = 0.883, *ω*^2^ < 0.001	BF_01_ = 3.161		
STAI trait	*F*(1,45) = 1.269, *p* = 0.266, *ω*^2^ = 0.005	BF_01_ = 2.037	*F*(1,49) = 1.288, *p* = 0.262, *ω*^2^ = 0.005	BF_01_ = 3.440		
Behavioral measures assessing executive control function						
Stop signal task	*F*(1,48) = 1.077, *p* = 0.304, *ω*^2^ = 0.002	BF_01_ = 2.278	*F*(1,48) = 0.067, *p* = 0.797, *ω*^2^ < 0.001	BF_01_ = 3.400		
Multi-source interference task	*F*(1,41) = 0.361, *p* = 0.552, *ω*^2^ < 0.001	BF_01_ = 2.934	*F*(1,40) = 0.214, *p* = 0.646, *ω*^2^ < 0.001	BF_01_ = 3.451		
TS switching costs	*F*(1,41) = 0.820, *p* = 0.370, *ω*^2^ < 0.001	BF_01_ = 2.383	*F*(1,41) = 2.169, *p* = 0.148, *ω*^2^ = 0.022	BF_01_ = 2.552		
TS mixing costs	*F*(1,41) = 0.013, *p* = 0.909, *ω*^2^ < 0.001	BF_01_ = 3.838	*F*(1,41) = 0.588, *p* = 0.447, *ω*^2^ < 0.001	BF_01_ = 1.883		
Summary statistics						
Harmonic mean		BF_01_ = 1.211		BF_01_ = 2.376		
Arithmetic mean	*F* = 0.940, *p* = 0.516, *ω*^2^ = 0.008	BF_01_ = 2.551	*F* = 0.866, *p* = 0.523, *ω*^2^ = 0.007	BF_01_ = 2.791		

*BP* Buss–Perry Aggression Questionnaire, *SHS* State Hostility Scale, *IRMAS* Updated Illinois Rape Myth Acceptance Scale, *RPFT* Rosenzweig Picture Frustration Test, *WVM* World View Measure, *BSSS* Brief Sensation Seeking Scale, *IRI* Interpersonal Reactivity Index, *ICQ* Interpersonal Competence Questionnaire, *RCRQ* Richardson Conflict Response Questionnaire, *STAI* State Trait Anxiety Inventory, *TS* Task switching, *ω*^2^ = partial omega square effect size measure (in the arithmetic mean of the partial omega square effect size <0.001 was treated as 0.001)

**Table 3:** Results of the statistical analysis using a classical frequentist approach and a Bayesian approach. Reported are the results of the group × time interaction while focusing on effects between baseline and posttest 2 and controlling for the confounding variables age and sex.

Dependent variables	GTA vs. Sims		GTA vs. Controls	
	Classical frequentist approach	Bayesian approach	Classical frequentist approach	Bayesian approach
Questionnaires assessing aggression and associated constructs				
BP physical aggression	*F*(1,35) = 0.486, *p* = 0.490, *ω*^2^ < 0.001	BF_01_ = 2.612	*F*(1,37) = 3.647, *p* = 0.064, *ω*^2^ = 0.063	BF_01_ = 0.597
BP verbal aggression	*F*(1,35) = 0.057, *p* = 0.813, *ω*^2^ < 0.001	BF_01_ = 3.113	*F*(1,37) = 0.342, *p* = 0.562, *ω*^2^ < 0.001	BF_01_ = 2.023
BP anger	*F*(1,35) = 0.511, *p* = 0.480, *ω*^2^ < 0.001	BF_01_ = 2.599	*F*(1,37) = 1.120, *p* = 0.297, *ω*^2^ = 0.003	BF_01_ = 1.754
BP hostility	*F*(1,35) = 1.193, *p* = 0.282, *ω*^2^ = 0.005	BF_01_ = 1.902	*F*(1,37) = 0.000, *p* = 0.998, *ω*^2^ < 0.001	BF_01_ = 3.252
SHS feeling mean	*F*(1,35) = 0.710, *p* = 0.405, *ω*^2^ < 0.001	BF_01_ = 2.390	*F*(1,37) = 0.245, *p* = 0.624, *ω*^2^ < 0.001	BF_01_ = 2.829
SHS aggravation	*F*(1,35) = 0.969, *p* = 0.332, *ω*^2^ < 0.001	BF_01_ = 2.090	*F*(1,37) = 0.262, *p* = 0.612, *ω*^2^ < 0.001	BF_01_ = 3.208
SHS feeling unsociable	*F*(1,35) = 0.619, *p* = 0.437, *ω*^2^ < 0.001	BF_01_ = 2.460	*F*(1,37) = 0.205, *p* = 0.653, *ω*^2^ < 0.001	BF_01_ = 3.128
SHS lack of positive feelings	*F*(1,35) = 0.495, *p* = 0.486, *ω*^2^ < 0.001	BF_01_ = 2.734	*F*(1,37) = 0.528, *p* = 0.472, *ω*^2^ < 0.001	BF_01_ = 2.183
IRMAS she asked for it	*F*(1,35) = 0.073, *p* = 0.789, *ω*^2^ < 0.001	BF_01_ = 2.974	*F*(1,37) = 0.000, *p* = 0.993, *ω*^2^ < 0.001	BF_01_ = 2.993
IRMAS he did not mean to	*F*(1,35) = 0.258, *p* = 0.615, *ω*^2^ < 0.001	BF_01_ = 2.964	*F*(1,37) = 0.011, *p* = 0.918, *ω*^2^ < 0.001	BF_01_ = 3.267
IRMAS it was not really rape	*F*(1,35) = 0.022, *p* = 0.883, *ω*^2^ < 0.001	BF_01_ = 3.369	*F*(1,37) = 0.889, *p* = 0.352, *ω*^2^ < 0.001	BF_01_ = 2.590
IRMAS she lied	*F*(1,35) = 0.385, *p* = 0.539, *ω*^2^ < 0.001	BF_01_ = 2.460	*F*(1,37) = 1.242, *p* = 0.272, *ω*^2^ = 0.005	BF_01_ = 2.363
Moral Disengagement Scale	*F*(1,33) = 1.001, *p* = 0.324, *ω*^2^ < 0.001	BF_01_ = 2.480	*F*(1,33) = 0.005, *p* = 0.945, *ω*^2^ < 0.001	BF_01_ = 2.962
RPFT extra-punitive	*F*(1,33) = 0.501, *p* = 0.484, *ω*^2^ < 0.001	BF_01_ = 2.436	*F*(1,30) = 0.358, *p* = 0.554, *ω*^2^ < 0.001	BF_01_ = 2.977
RPFT intra-punitive	*F*(1,32) = 0.705, *p* = 0.407, *ω*^2^ < 0.001	BF_01_ = 2.010	*F*(1,31) = 0.027, *p* = 0.870, *ω*^2^ < 0.001	BF_01_ = 2.666
RPFT non-punitive	*F*(1,32) = 0.782, *p* = 0.383, *ω*^2^ < 0.001	BF_01_ = 1.582	*F*(1,32) = 4.328, *p* = 0.046*, *ω*^2^ = 0.063	BF_01_ = 0.700
RPFT obstacle dominance	*F*(1,32) = 0.110, *p* = 0.742, *ω*^2^ < 0.001	BF_01_ = 3.218	*F*(1,31) = 0.124, *p* = 0.727, *ω*^2^ < 0.001	BF_01_ = 2.900
RPFT ego-defense	*F*(1,33) = 1.781, *p* = 0.191, *ω*^2^ = 0.017	BF_01_ = 0.773	*F*(1,31) = 6.115, *p* = 0.019*, *ω*^2^ = 0.102	BF_01_ = 0.328
RPFT need persistence	*F*(1,32) = 0.409, *p* = 0.527, *ω*^2^ < 0.001	BF_01_ = 2.328	*F*(1,31) = 0.567, *p* = 0.457, *ω*^2^ < 0.001	BF_01_ = 2.759
WVM percent	*F*(1,35) = 0.669, *p* = 0.419, *ω*^2^ < 0.001	BF_01_ = 2.315	*F*(1,37) = 1.468, *p* = 0.233, *ω*^2^ = 0.012	BF_01_ = 2.018
WVM safety	*F*(1,35) = 0.150, *p* = 0.701, *ω*^2^ < 0.001	BF_01_ = 3.548	*F*(1,37) = 0.064, *p* = 0.801, *ω*^2^ < 0.001	BF_01_ = 3.058
Behavioral measures assessing aggression				
Word completion test	*F*(1,35) = 6.844, *p* = 0.013*, *ω*^2^ = 0.086	BF_01_ = 0.161	*F*(1,35) = 1.792, *p* = 0.189, *ω*^2^ = 0.022	BF_01_ = 1.717
Lexical decision task	*F*(1,47) = 0.002, *p* = 0.962, *ω*^2^ < 0.001	BF_01_ = 3.618	*F*(1,50) = 0.009, *p* = 0.926, *ω*^2^ < 0.001	BF_01_ = 3.522
Delay frustration task	*F*(1,37) = 1.626, *p* = 0.210, *ω*^2^ = 0.016	BF_01_ = 1.361	*F*(1,40) = 1.471, *p* = 0.232, *ω*^2^ = 0.010	BF_01_ = 1.695
Sensation seeking and boredom proneness				
BSSS experience seeking	*F*(1,35) = 0.928, *p* = 0.342, *ω*^2^ < 0.001	BF_01_ = 2.327	*F*(1,37) = 2.988, *p* = 0.092, *ω*^2^ = 0.048	BF_01_ = 0.924
BSSS boredom susceptibility	*F*(1,35) = 0.297, *p* = 0.589, *ω*^2^ < 0.001	BF_01_ = 3.022	*F*(1,37) = 3.559, *p* = 0.067, *ω*^2^ = 0.058	BF_01_ = 0.493
BSSS thrill and adventure seeking	*F*(1,35) = 0.000, *p* = 0.996, *ω*^2^ < 0.001	BF_01_ = 3.280	*F*(1,37) = 0.386, *p* = 0.538, *ω*^2^ < 0.001	BF_01_ = 2.714
BSSS disinhibition	*F*(1,35) = 0.502, *p* = 0.483, *ω*^2^ < 0.001	BF_01_ = 2.673	*F*(1,36) = 0.073, *p* = 0.789, *ω*^2^ < 0.001	BF_01_ = 3.363
Boredom proneness scale	*F*(1,35) = 0.052, *p* = 0.820, *ω*^2^ < 0.001	BF_01_ = 3.205	*F*(1,37) = 0.400, *p* = 0.531, *ω*^2^ < 0.001	BF_01_ = 2.028
Behavioral measures assessing risk taking and delay discounting				
Balloon analogue risk task	*F*(1,47) = 0.471, *p* = 0.471, *ω*^2^ < 0.001	BF_01_ = 2.873	*F*(1,47) = 0.420, *p* = 0.520, *ω*^2^ < 0.001	BF_01_ = 2.665
Delay-discounting task delay	*F*(1,45) = 0.646, *p* = 0.426, *ω*^2^ < 0.001	BF_01_ = 2.429	*F*(1,49) = 0.387, *p* = 0.537, *ω*^2^ < 0.001	BF_01_ = 2.346
Delay-discounting task probability	*F*(1,45) = 0.347, *p* = 0.559, *ω*^2^ < 0.001	BF_01_ = 2.838	*F*(1,49) = 1.131, *p* = 0.293, *ω*^2^ = 0.003	BF_01_ = 1.735
Questionnaires and tests assessing empathy and interpersonal competence				
IRI perspective taking	*F*(1,36) = 1.637, *p* = 0.209, *ω*^2^ = 0.015	BF_01_ = 1.493	*F*(1,38) = 1.636, *p* = 0.209, *ω*^2^ = 0.015	BF_01_ = 2.041
IRI fantasy scale	*F*(1,56) = 0.447, *p* = 0.507, *ω*^2^ < 0.001	BF_01_ = 2.651	*F*(1,56) = 0.154, *p* = 0.696, *ω*^2^ < 0.001	BF_01_ = 3.341
IRI empathic concern	*F*(1,36) = 0.027, *p* = 0.871, *ω*^2^ < 0.001	BF_01_ = 3.006	*F*(1,38) = 0.288, *p* = 0.594, *ω*^2^ < 0.001	BF_01_ = 3.162
IRI personal distress	*F*(1,56) = 0.029, *p* = 0.867, *ω*^2^ < 0.001	BF_01_ = 3.915	*F*(1,56) = 0.099, *p* = 0.754, *ω*^2^ < 0.001	BF_01_ = 3.585
Balanced emotional empathy scale	*F*(1,34) = 2.293, *p* = 0.139, *ω*^2^ = 0.033	BF_01_ = 1.335	*F*(1,36) = 0.076, *p* = 0.785, *ω*^2^ < 0.001	BF_01_ = 3.025
Reading the mind in the eyes	*F*(1,33) = 1.005, *p* = 0.636, *ω*^2^ < 0.001	BF_01_ = 2.964	*F*(1,33) = 1.019, *p* = 0.626, *ω*^2^ < 0.001	BF_01_ = 2.746
ICQ initiating relationships	*F*(1,35) = 1.761, *p* = 0.193, *ω*^2^ = 0.017	BF_01_ = 2.003	*F*(1,37) = 1.163, *p* = 0.288, *ω*^2^ = 0.004	BF_01_ = 2.180
ICQ negative assertion	*F*(1,35) = 3.352, *p* = 0.076, *ω*^2^ = 0.037	BF_01_ = 0.797	*F*(1,37) = 0.643, *p* = 0.428, *ω*^2^ < 0.001	BF_01_ = 2.557
ICQ disclosing personal information	*F*(1,35) = 0.337, *p* = 0.565, *ω*^2^ < 0.001	BF_01_ = 2.413	*F*(1,37) = 3.335, *p* = 0.076, *ω*^2^ = 0.051	BF_01_ = 1.491
ICQ providing emotional support	*F*(1,35) = 1.032, *p* = 0.317, *ω*^2^ = 0.001	BF_01_ = 1.691	*F*(1,37) = 1.389, *p* = 0.246, *ω*^2^ = 0.007	BF_01_ = 2.076
ICQ advice and managing social conflict	*F*(1,35) = 0.001, *p* = 0.970, *ω*^2^ < 0.001	BF_01_ = 3.086	*F*(1,37) = 0.005, *p* = 0.943, *ω*^2^ < 0.001	BF_01_ = 3.197
RCRQ direct aggression	*F*(1,35) = 0.184, *p* = 0.671, *ω*^2^ < 0.001	BF_01_ = 2.936	*F*(1,37) = 0.106, *p* = 0.746, *ω*^2^ < 0.001	BF_01_ = 2.848
RCRQ indirect aggression	*F*(1,35) = 0.325, *p* = 0.572, *ω*^2^ < 0.001	BF_01_ = 2.948	*F*(1,37) = 2.227, *p* = 0.144, *ω*^2^ = 0.029	BF_01_ = 1.264
Questionnaires assessing depressivity and anxiety				
Beck depression inventory	*F*(1,35) = 0.167, *p* = 0.685, *ω*^2^ < 0.001	BF_01_ = 2.768	*F*(1,37) = 0.400, *p* = 0.531, *ω*^2^ < 0.001	BF_01_ = 2.756
STAI state	*F*(1,35) = 0.219, *p* = 0.643, *ω*^2^ < 0.001	BF_01_ = 2.834	*F*(1,38) = 1.089, *p* = 0.303, *ω*^2^ = 0.002	BF_01_ = 2.140
STAI trait	*F*(1,35) = 0.219, *p* = 0.642, *ω*^2^ < 0.001	BF_01_ = 2.560	*F*(1,38) = 0.263, *p* = 0.611, *ω*^2^ < 0.001	BF_01_ = 3.305
Behavioral measures assessing executive control function				
Stop signal task	*F*(1,47) = 0.976, *p* = 0.328, *ω*^2^ < 0.001	BF_01_ = 2.139	*F*(1,47) = 0.000, *p* = 0.985, *ω*^2^ < 0.001	BF_01_ = 3.423
Multi-source interference task	*F*(1,38) = 0.157, *p* = 0.694, *ω*^2^ < 0.001	BF_01_ = 3.167	*F*(1,37) = 0.544, *p* = 0.465, *ω*^2^ < 0.001	BF_01_ = 3.011
TS switching costs	*F*(1,38) = 0.993, *p* = 0.325, *ω*^2^ < 0.001	BF_01_ = 1.781	*F*(1,37) = 0.019, *p* = 0.892, *ω*^2^ < 0.001	BF_01_ = 1.911
TS mixing costs	*F*(1,38) = 10.373, *p* = 0.003*, *ω*^2^ = 0.184	BF_01_ = 0.067	*F*(1,37) = 1.683, *p* = 0.203, *ω*^2^ = 0.017	BF_01_ = 1.179
Summary statistics				
Harmonic mean		BF_01_ = 1.380		BF_01_ = 1.808
Arithmetic mean	*F* = 0.945, *p* = 0.510, *ω*^2^ = 0.009	BF_01_ = 1.201	*F* = 0.967, *p* = 0.514, *ω*^2^ = 0.011	BF_01_ = 2.404

*BP* Buss–Perry Aggression Questionnaire, *SHS* State Hostility Scale, *IRMAS* Updated Illinois Rape Myth Acceptance Scale, *RPFT* Rosenzweig Picture Frustration Test, *WVM* World View Measure, *BSSS* Brief Sensation Seeking Scale, *IRI* Interpersonal Reactivity Index, *ICQ* Interpersonal Competence Questionnaire, *RCRQ* Richardson Conflict Response Questionnaire, *STAI* State Trait Anxiety Inventory, *TS* Task switching, *ω*^2^ = partial omega square effect size measure (in the arithmetic mean of the partial omega square effect size <0.001 was treated as 0.001)

Since we conducted 208 separate frequentist tests we expected 10.4 significant effects simply by chance when setting the alpha value to 0.05. In fact we found only eight significant time × group interactions (these are marked with an asterisk in Tables 2 and 3).

When applying a conservative Bonferroni correction, none of those tests survive the corrected threshold of *p* < 0.00024. Neither does any test survive the more lenient FDR correction. The arithmetic mean of the frequentist test statistics likewise shows that on average no significant effect was found (bottom rows in Tables 2 and 3).

In line with the findings from a frequentist approach, the harmonic mean of the Bayesian factor BF_01_ is consistently above one but not very far from one. This likewise suggests that there is very likely no interaction between group × time and therewith no detrimental effects of the violent video game GTA in the domains tested. The evidence in favour of the null hypothesis based on the Bayes factor is not massive, but clearly above 1. Some of the harmonic means are above 1.6 and constitute substantial evidence [48]. However, the harmonic mean has been criticised as unstable. Owing to the fact that the sum is dominated by occasional small terms in the likelihood, one may underestimate the actual evidence in favour of the null hypothesis [49].

To test the sensitivity of the present study to detect relevant effects we computed the effect size that we would have been able to detect. The information we used consisted of alpha error probability = 0.05, power = 0.95, our sample size, number of groups and of measurement occasions and correlation between the repeated measures at posttest 1 and posttest 2 (average *r* = 0.68). According to G*Power [50], we could detect small effect sizes of *f* = 0.16 (equals *η*^2^ = 0.025 and *r* = 0.16) in each separate test. When accounting for the conservative Bonferroni-corrected *p*-value of 0.00024, still a medium effect size of *f* = 0.23 (equals *η*^2^ = 0.05 and *r* = 0.22) would have been detectable. A meta-analysis by Anderson [2] reported an average effects size of *r* = 0.18 for experimental studies testing for aggressive behaviour and another by Greitmeyer [5] reported average effect sizes of *r* = 0.19, 0.25 and 0.17 for effects of violent games on aggressive behaviour, cognition and affect, all of which should have been detectable at least before multiple test correction.

## Discussion

Within the scope of the present study we tested the potential effects of playing the violent video game *GTA V* for 2 months against an active control group that played the non-violent, rather pro-social life simulation game The *Sims 3* and a passive control group. Participants were tested before and after the long-term intervention and at a follow-up appointment 2 months later. Although we used a comprehensive test battery consisting of questionnaires and computerised behavioural tests assessing aggression, impulsivity-related constructs, mood, anxiety, empathy, interpersonal competencies and executive control functions, we did not find relevant negative effects in response to violent video game playing. In fact, only three tests of the 208 statistical tests performed showed a significant interaction pattern that would be in line with this hypothesis. Since at least ten significant effects would be expected purely by chance, we conclude that there were no detrimental effects of violent video gameplay.

This finding stands in contrast to some experimental studies, in which short-term effects of violent video game exposure have been investigated and where increases in aggressive thoughts and affect as well as decreases in helping behaviour have been observed [1]. However, these effects of violent video gaming on aggressiveness—if present at all (see above)—seem to be rather short-lived, potentially lasting <15 min [8, 51]. In addition, these short-term effects of video gaming are far from consistent as multiple studies fail to demonstrate or replicate them [16, 17]. This may in part be due to problems, that are very prominent in this field of research, namely that the outcome measures of aggression and pro-social behaviour, are poorly standardised, do not easily generalise to real-life behaviour and may have lead to selective reporting of the results [3]. We tried to address these concerns by including a large set of outcome measures that were mostly inspired by previous studies demonstrating effects of short-term violent video gameplay on aggressive behaviour and thoughts, that we report exhaustively.

Since effects observed only for a few minutes after short sessions of video gaming are not representative of what society at large is actually interested in, namely how habitual violent video gameplay affects behaviour on a more long-term basis, studies employing longer training intervals are highly relevant. Two previous studies have employed longer training intervals. In an online study, participants with a broad age range (14–68 years) have been trained in a violent video game for 4 weeks [52]. In comparison to a passive control group no changes were observed, neither in aggression-related beliefs, nor in aggressive social interactions assessed by means of two questions. In a more recent study, participants played a previous version of GTA for 12 h spread across 3 weeks [53]. Participants were compared to a passive control group using the Buss–Perry aggression questionnaire, a questionnaire assessing impulsive or reactive aggression, attitude towards violence, and empathy. The authors only report a limited increase in pro-violent attitude. Unfortunately, this study only assessed posttest measures, which precludes the assessment of actual changes caused by the game intervention.

The present study goes beyond these studies by showing that 2 months of violent video gameplay does neither lead to any significant negative effects in a broad assessment battery administered directly after the intervention nor at a follow-up assessment 2 months after the intervention. The fact that we assessed multiple domains, not finding an effect in any of them, makes the present study the most comprehensive in the field. Our battery included self-report instruments on aggression (Buss–Perry aggression questionnaire, State Hostility scale, Illinois Rape Myth Acceptance scale, Moral Disengagement scale, World View Measure and Rosenzweig Picture Frustration test) as well as computer-based tests measuring aggressive behaviour such as the delay frustration task and measuring the availability of aggressive words using the word completion test and a lexical decision task. Moreover, we assessed impulse-related concepts such as sensation seeking, boredom proneness and associated behavioural measures such as the computerised Balloon analogue risk task, and delay discounting. Four scales assessing empathy and interpersonal competence scales, including the reading the mind in the eyes test revealed no effects of violent video gameplay. Neither did we find any effects on depressivity (Becks depression inventory) nor anxiety measured as a state as well as a trait. This is an important point, since several studies reported higher rates of depressivity and anxiety in populations of habitual video gamers [54, 55]. Last but not least, our results revealed also no substantial changes in executive control tasks performance, neither in the Stop signal task, the Multi-source interference task or a Task switching task. Previous studies have shown higher performance of habitual action video gamers in executive tasks such as task switching [56–58] and another study suggests that training with action video games improves task performance that relates to executive functions [59], however, these associations were not confirmed by a meta-analysis in the field [60]. The absence of changes in the stop signal task fits well with previous studies that likewise revealed no difference between in habitual action video gamers and controls in terms of action inhibition [61, 62]. Although GTA does not qualify as a classical first-person shooter as most of the previously tested action video games, it is classified as an action-adventure game and shares multiple features with those action video games previously related to increases in executive function, including the need for hand–eye coordination and fast reaction times.

Taken together, the findings of the present study show that an extensive game intervention over the course of 2 months did not reveal any specific changes in aggression, empathy, interpersonal competencies, impulsivity-related constructs, depressivity, anxiety or executive control functions; neither in comparison to an active control group that played a non-violent video game nor to a passive control group. We observed no effects when comparing a baseline and a post-training assessment, nor when focussing on more long-term effects between baseline and a follow-up interval 2 months after the participants stopped training. To our knowledge, the present study employed the most comprehensive test battery spanning a multitude of domains in which changes due to violent video games may have been expected. Therefore the present results provide strong evidence against the frequently debated negative effects of playing violent video games. This debate has mostly been informed by studies showing short-term effects of violent video games when tests were administered immediately after a short playtime of a few minutes; effects that may in large be caused by short-lived priming effects that vanish after minutes. The presented results will therefore help to communicate a more realistic scientific perspective of the real-life effects of violent video gaming. However, future research is needed to demonstrate the absence of effects of violent video gameplay in children.

## Electronic supplementary material

Supplementary Material
