# Supplementary material for: Does playing violent video games cause aggression? A longitudinal intervention study
Source: Mol Psychiatry. 2018 Mar 13;24(8):1220–34. doi: 10.1038/s41380-018-0031-7 (PMC6756088; doi:10.1038/s41380-018-0031-7)
Supplement: Supplementary file 1 — Supplementary Material [file 41380_2018_31_MOESM1_ESM.pdf]

Supplementary Material

Questionnaires assessing aggression and associated constructs

Buss-Perry Aggression Questionnaire (BP) [1]. A measure of trait aggressiveness consisting of 27 items with a 7-point response scale ranging from “very characteristic” to “very uncharacteristic” with four subscales of physical and verbal aggression, anger and hostility. Cronbach’s alpha= 0.89, test-retest reliability r=0.80 [1].

State Hostility Scale (SHS) [2]. The SHS consists of 35 items with a 5-point response scale ranging from “strongly agree” to “strongly disagree” and has been shown to increase under acute situational variables such as pain, provocation, violent movie clips and uncomfortably cold and hot temperatures. It consists of the four subscales feeling mean, aggravation, feeling unsociable and lack of positive feelings [3]. Cronbach’s alpha= 0.95 [4].

Updated Illinois Rape Myth Acceptance Scale (IRMAS) [5, 6]. The IRMAS was used to assess rape myth acceptance with 22 items on a 5-point response scale ranging from “strongly agree” to “strongly disagree”. The following four subscales were computed “she asked for it”, “he didn’t mean to”, “it wasn’t really rape” and “she lied”. Cronbach’s alpha= 0.87, test-retest reliability=0.87 [7].

Moral Disengagement Scale (MDS) [8, 9]. The MDS recurs to the social cognitive theory presented by Bandura [10]. His theory poses that usually self-regulatory processes deter transgressive behaviour through self-condemnation that individuals anticipate were they to engage in behaviour that is in conflict with their internalized moral standards. Moral disengagement explains how this self-regulatory processes can fail. The MDS consists of 8 items and we used a 5-point response scale ranging from “strongly disagree” to “strongly agree”. Cronbach’s alpha= 0.76 [11].

Rosenzweig Picture Frustration Test (RPFT) [12, 13]. The RPFT is a so-called projective test of aggression and consists of 24 cartoon pictures each portraying persons in a frustrating situation (e.g. getting wet by a car driving through a puddle of water). Each picture contains two speech balloons, one filled by a frustrator and a blank one for the participant to fill in with the first response that comes to mind. The responses are scored in relation to psychological defense mechanism. As suggested by Rosenzweig we discriminated between three response directions of aggression by the frustrated individual: extra-punitive (E) in which the aggression was directed outwards. In this case, something or someone was found

guilty by the frustrated individual; intra-punitive (I), in which the aggression was directed towards the participant him/herself and non-punitive response (M), wherein the aggression was avoided, and the frustrating situation was described as insignificant, without fault, or as susceptible of being improved. The type of reaction was divided according to three categories: obstacle-dominance (OD), in which the obstacle that causes frustration is mentioned and emphasized by the participant, ego-defense (ED), in which the individual either blames others or accepts responsibility, or even states that the responsibility of the situation is no more fit; need-persistence (NP), in which the trend of response is directed towards solving the problem inherent to the frustrating situation. The RPFT was rated by two experts showing high inter-rater reliability (0.917). Disagreement between the judgements of the two experts were discussed until they found a mutually satisfactory solution.

World View Measure (WVM) [14] consisting of four items asking for the likelihood (in percent) of different crimes taking place and two items asking about the feelings of safety on a 7-point rating scale ranging from "not save at all" to "absolutely save".

Behavioral measures assessing aggression

Word Completion Task (WCT). The WCT presents word fragments to the participants that they need to complete so that a real word results. The task has been used in English in previous studies assessing the short-term effects of violent video games [15]. We created a German version of a task consisting of 100 words and gave participants 5 min to complete the list. Participants were allowed to skip items that they were not able to solve. We computed an index that captures the propensity to think of aggression-related concepts. For this we divided the number of aggression-related words by the number of actual words that the participants completed (we excluded words that were not part of the German language).

Lexical Decision Task (LDT) [16]. We used a LDT to evaluate how readily accessible aggressive thoughts were. The objective of this task was to evaluate how quickly participants could identify words linked to aggression relative to neutral words. Participants completed 112 trials, the first 12 of which were practice trials. Each trial began with participants focusing on a white fixation cross for 1000 ms, which was replaced by a string of letters that was either a legitimate or a plausible but illegitimate German word presented in white on a black background. Participants were instructed to tap the spacebar as quickly as possible if the string was a legitimate German word and to wait until the next trial began if the string was a nonword (3000 ms). Six practice trials presented neutral words, and five used text strings of

plausible nonwords. The remaining trials consisted of 56 strings that were nonwords, 28 neutral verbs, and 28 verbs related to aggression (e.g., killing, kicking, hurting). Reaction times to legitimate German verbs below 200 ms and above 2000 ms were discarded as outliers. These dropped cases comprised less than 2% of all trials. There were no significant condition differences in word accuracy between conditions. Aggressive thought scores were calculated for each participant by subtracting reaction times to aggression-related verbs from reaction times to neutral verbs.

Delay frustration task (DeFT) [17]. The DeFT is a measure of delay intolerance. Participants perform a series of simple math questions presented on a computer which have to be answered by pressing a designated response button. On selected trials a delay is inserted in which the task stops. These delay periods are unpredictable and unsignalled but interrupt the completion of the task. Delay frustration is indexed as the number and duration of responses made on a response key during these interruptions. We used the number of key presses during these delay phases as a measure of delay frustration.

Sensation seeking and boredom proneness

Brief Sensation Seeking Scale (BSSS) [18]. The BSSS consists of 8 items to which participants responded on a 5-point rating scale ranging from „strongly disagree“ to „strongly agree“. It includes the subscales experience seeking (ES), boredom susceptibility (BS), thrill and adventure seeking (TAS), and disinhibition (DI). Cronbach’s alpha = 0.76.

Boredom Propensity Scale (BPS) [19]. The BPS measures the propensity to become bored and has been closely related to the propensity to become depressed and pursue thrill-seeking behaviour. It consists of 28 items, which have to be responded to on a 7-point rating scale ranging from “strongly disagree” to “strongly agree”. Cronbach’s alpha= 0.79, Test-retest reliability r=0.83.

Behavioral measures assessing risk taking and delay-discounting

Balloon Analogue Risk Task (BART) [20]. The BART is a computerized measure of risk taking behaviour. Participants are presented with a balloon and offered the chance to earn money by pumping the balloon up by pressing a button. Each button press causes the balloon to incrementally inflate and money to be added to an account. However at some point the balloon is overinflated and explodes. Therefore each pump confers greater risk but also

greater potential reward. If the participant chooses to cash-out prior to the explosion, they collect the money for this trial; if the balloon explodes the earnings for this trial are lost. Participants were not informed about the balloons breakpoints. At the end of the task participants received ¼ of the amount that they had won (on average 4.50 Euros, ranging from 3.50-6.00 Euros). The primary score used to measure risk taking is the adjusted average number of pumps on unexploded balloons, with higher scores indicative of greater risk-taking propensity.

Delay Discounting Task (DDT) [21]. In this computerized DDT task subjects were presented with 84 hypothetical choices between a smaller, immediate reward and a larger reward (10 €) that was delayed by different intervals (0, 2, 30, 180, or 365 days). On each of these trials participants were required to indicate their preference for one out of the two-presented options by pressing a spatially corresponding button on the keyboard (e.g. "Would you rather have, say, 8 € now or 10 € in, say, 30 days?"). At each delay to the standard reward, an indifference or switch point was identified using a random adjusting-amount procedure. These indifference points highlight the point of immediate money at which an individual judges the money to be subjectively equivalent to the larger amount that is delayed. In order to ensure that participants consider their monetary choices seriously we told them that they receive money based on one random choice divided by three (participants were paid on average 2.50 Euros, ranging from 1.00-3.50 Euros).

The operational definition of impulsivity inherent in the DDT states that impulsivity is revealed by a preference for smaller, more immediate rewards over larger, more delayed rewards [22]. Hence, the value of a delayed reward is discounted in inverse proportion to its delay, so that the more distal the reward the less the value that is ascribed to its receipt. Typically individuals with a higher degree of impulsivity tend to discount the value of a relatively delayed reward considerably stronger than the presented smaller and immediate alternative. Therefore, the steepness of the discounting function fitted to the five obtained indifference points discloses an individual's amount of delay discounting which is an indicator for impulsivity. A steeper decrease in value as reflected in a larger steepness parameter k is indicative of a higher degree of impulsivity. Research on delay discounting has identified a hyperbolic function to be the best fit to discounting data [23]. In the hyperbolic function, $V = A / (1 + kD)$. V represents the present value of a reward or in other words the indifference point, which is equal to the monetary amount of the reward (A) divided by the delay (D) to the reward's receipt. The numeral 1 in the denominator ensures that the value does not approach infinity as the delay approaches 0. The most essential part of the equation

is the free parameter *k*, which expresses how much value is affected by delay. If *k* is fairly large, this will mean that that the effect of delay (D) on discounting value is bigger than if *k* is small. Hence, higher values of *k* indicate a preference for smaller, immediate rewards and is characteristic of impulsive individuals. In order to obtain *k* values for each individual at each point of measurement, a hyperbolic function has been fitted to an individual's indifference points using IPython notebook and a least-square fit function.

Questionnaires and tests assessing empathy and interpersonal competence

Interpersonal Reactivity Index (IRI) [24]. The IRI is a commonly used measure to assess empathy consisting of 28 items with a 5-point response scale ranging from "does not apply at all" to "totally applies". It contains the four subscales perspective taking, fantasy, empathic concern and personal distress. Cronbach's alpha= 0.71-0.78, Test-retest reliability= 0.62-0.80

Balanced Emotional Empathy Scale (BEES) [25]. The BEES assesses how well individuals are able to feel the emotional experiences of others assuming that this immediate feedback discourages aggressive acts and it has been shown to relate negatively to interpersonal violence. In the present study, it consists of 30 items to which participants responded on a 7-point rating scale, ranging from "does not apply at all" to "totally applies". Cronbach's alpha= 0.87, Test-retest reliability r=0.77.

Reading the Mind in the Eyes (RME) [26]. The RME consists of 36 photographs of pairs of eyes that express an emotion. Four words characterizing the emotional state of the person of whom the photograph has been taken are printed around it and the participant has to select the most fitting state. Cronbach's alpha= 0.77, Test-retest reliability r=0.70 [27].

Interpersonal Competence Questionnaire (ICQ) [28]. The ICQ measures social competence and consists of 40 items assessing five subdomains of social competence namely initiating relationships, negative assertion, disclosing personal information, providing emotional support and advice and managing social conflict. Each items describes a social interaction and is rated on a 5-point rating scale to indicate the individual's level of competence and confidence in these situations (1 = "I'm poor at this; I'd feel so uncomfortable and unable to handle this situation, I'd avoid it if possible"; 2 = I'm only fair at this; I'd feel uncomfortable and have lots of difficulty handling this situation"; 3 = "I'm okay at this; I'd feel somewhat uncomfortable and have some difficulty handling this situation"; 4 = "I'm good at this; I'd feel quite comfortable and able to handle this situation"; 5 = "I'm extremely good at this; I'd

feel comfortable and could handle this situation very well"). Cronbach's alpha= 0.83, Test-retest reliability r=0.69-0.89.

Richardson Conflict Response Questionnaire (RCRQ) [29]. The RCRQ asks how frequently in the past month participants have engaged in the proposed aggressive behaviour. It discriminates between direct and indirect aggression levels (and contains filler items) and consists of 28 items with a 5-point response scale ranging from "never" to "very often".

Questionnaires assessing depressivity and anxiety

Beck Depression Inventory (BDI) [30]. BDI measures the severity of depression in 21-items each scored on a scale value from "0" to "3". Cronbach's alpha= 0.86, [31], Test-retest reliability (r=0.93) [32].

State Trait Anxiety Inventory (STAI) [33]. This inventory consists of two self-report scales; the first measures state anxiety ('Indicate how you feel right now'), and the second measures trait anxiety ('Indicate how you generally feel'). Cronbach's alpha =0.88-0.94.

Behavioral measures assessing executive control function

Stop Signal Task (SST) [34]. The computerized stop task consisted of trials in which a right or left button press was required (go trials) and trials in which an already initiated motor command had to be withheld (stop trials). Participants were instructed to respond as fast as possible to a white arrow pointing either to the right or left by pressing a right or left button (using right and left index finger, respectively). For stop trials (25% of trials), participants were instructed to stop their response when the white arrow changed its color to red after a certain delay (stop signal delay, SSD). The adaptive character of the task was achieved by a continuous adaption of the SSD using two independent staircases in order to reach a performance level of approximately 50% successfully inhibited responses. The staircases started at 150 and 200 ms, respectively. A staircase was incremented in case of a successful stop trial (no button press) or decremented in case of an unsuccessful stop trial (executed button press) by 50 ms. The mean SSD during the pretest was used as a starting value for the two staircases of the experiment. During the experiment, the SSD was also updated continuously in the same way as described for the pretest.

All trials started with a cue, which lasted for 500 ms; during this time a white circular ring was presented in the center of the screen. Subsequently, an arrow appeared in the center of the

ring either pointing to the right (>) or left (<). The direction of the arrow was balanced across the experiment in a pseudo-randomized order. A go trial (72 of 96 trials) ended in the moment of a button press, but lasted a maximum of 1000 ms. In the stop trials (24 of 96 trials) the arrow and the ring changed their color to red once the adaptive SSD had elapsed, indicating the response should be withheld. Stop trials also lasted a maximum of 1000 ms and ended previously, if the participant did not withhold the response. At the end of each trial, a blank black screen was presented.

Logan and Cowan suggested that go and the stop processes are two independent processes, which compete against each other [35]. When the probability of responding on a stop-signal trial is different from 0.50, the integration method to compute SSRT is recommended [36]. According to this method SSRT is estimated by subtracting mean SSD from the finishing time of the stop process, which is determined by integrating the Go RT distribution. The resulting SSRT is an estimation of the length of inhibition process and thus a measure of inhibition performance.

Multi-Source Interference Task (MSI) [37]. The computerized version of the MSI was composed of the numbers 0, 1, 2, or 3 presented in the center of the screen every few seconds. Participants were instructed that the target number would always be different from the other two numbers and their task was to report the identity of the target number via a button-press. Participants used their right index, middle and ring finger to respond. During the control condition, the target number always matched its position and was accompanied by two zeros in the two other positions (100, 020, 003). In the interference condition on the other hand, the target number never matched its position, and no zeros would be included; instead the distracters themselves would be potential targets (211, 221, 232). It was emphasized that the subjects should report the target number regardless of its position and that the subjects should answer as quickly as possible, but not sacrifice accuracy for speed. As an indicator for the interference effect we subtracted response times for correct control trials from correct interference trials.

Task Switching Task (TS) [38]. In this computerized TS experiment in each trial of the blocked explicitly cued task, coloured shapes were presented centrally on the monitor as target stimuli against a light grey background. Participants had to perform one of two possible tasks in each trial: the "colour" task or the "shape" task. The task to be performed on a given trial was indicated by a verbal cue printed in German. In colour task trials, participants had to decide whether a presented shape was red or blue by means of a button press. In shape task trials,

they had to classify the presented shape as a square or diamond. Correct responses were made by means of a left button press for a red target stimulus in colour trials and a square in shape trials. Correct responses were made by means of a right button press for a blue target stimulus in colour trials and a diamond in shape trials. The paradigm consisted of two types of task blocks: single task blocks (pure repetition blocks) and mixed task blocks. In pure repetition blocks, participants had to repeatedly perform exclusively either the colour task or the shape task. In mixed task blocks, participants had to alternate in an unpredictable manner between both of the tasks. We derived two measurements from the TS task, namely switch costs, which were computed as the difference in reaction time between switch and repetition trials in the mixed blocks and mixing costs, computed as the difference between repetition trials in the mixed blocks and trials from the pure repetition blocks.

Description of the three effects that reflected detrimental effects of violent video game play (but did not survive multiple test correction)

Only three of the 8 significnat measures actually display a pattern showing detrimental effects of violent video game play. The IRMAS item „she lied“ displayed a pattern where GTA participants showed a stronger increase between Baseline and Posttest 1 than Sims participants, and the Word completion task where GTA participants showed a stronger increase in the aggressive word ratio than Sims participants between Baseline and Posttest 1 as well as Posttest 2.

Inter-correlations between the different outcome measures of aggression

Figure 1: Inter-correlation matrix of the data of all participants (n=90) at Baseline. Red indicates positive, blue negative Pearson correlation coefficients. BP = Buss-Perry Aggression Questionnaire, SHS = State Hostility Scale, IRMAS = Updated Illinois Rape Myth Acceptance Scale, WVM = World View Measure, PMDS = Moral Disengagement Scale, RPFT = Rosenzweig Picture Frustration Test.

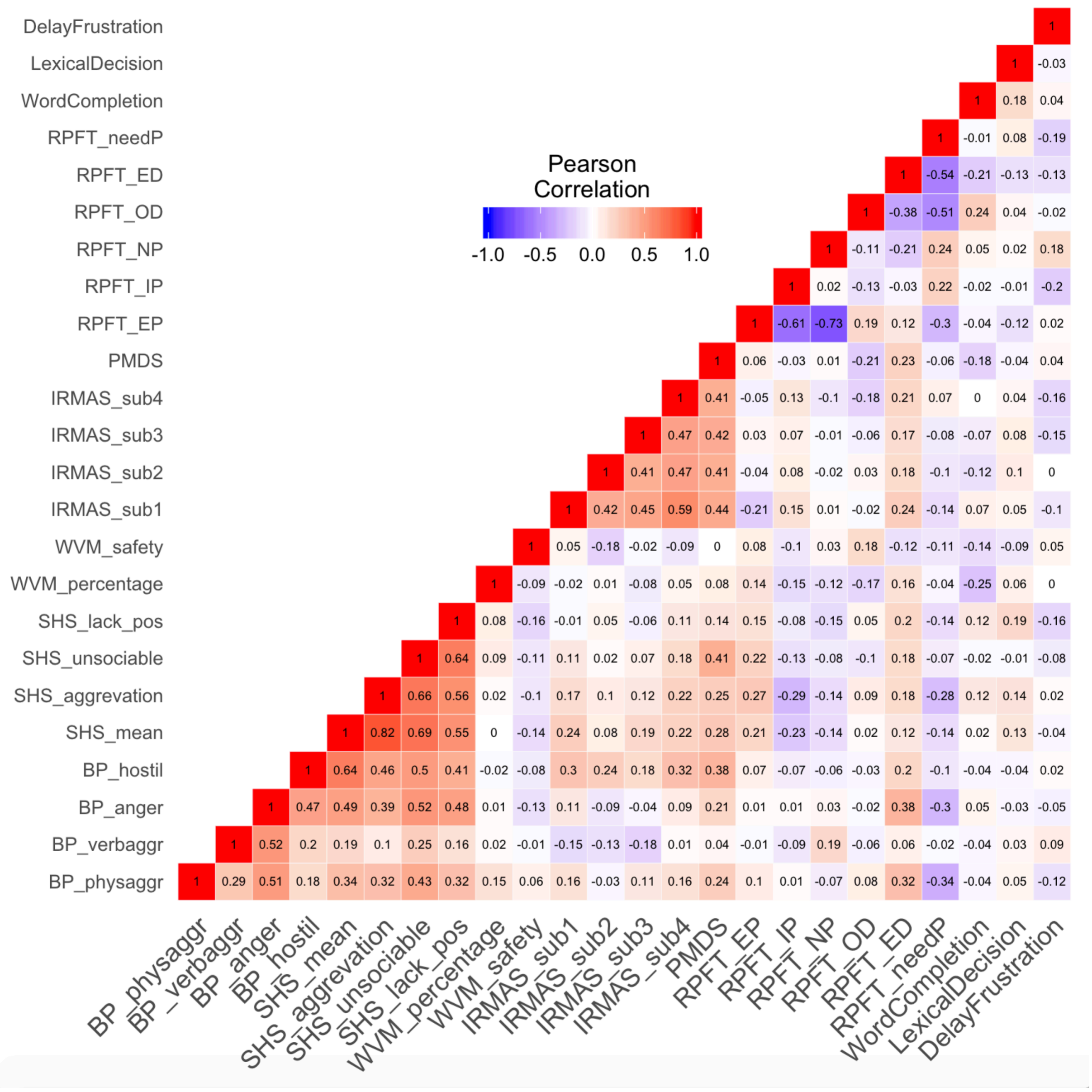

Latent factor modelling

The outcome measures were carefully chosen with the goal to built latent factors and assess potential changes due to the intervention on a latent level. Concerning the latent factor of aggression we have been unsuccessful to built stable latent factors, a fact that is reflected in the low inter-correlations between the outcome measures of aggression (see above). However, we do think that this is due to the construct of aggression since other constructs such as impulsivity and empathy did result in stable latent factors on pretest data in the present study.

Our theory-driven latent impulsivity factor, consisted of the following manifest indicators: Boredom Proneness total score, Brief Sensation Seeking Scale total score, Delay discounting probability, the adjusted average number of pumps on unexploded balloons in the Balloon Analogue Risk Task. The commonly used indicators in a structural equation modelling framework indicated good model fit: Root Mean Square Error of Approximation (RMSEA)<0.001, 90% confidence interval: 0.000-0.164, Comparative Fit Index (CFI) =1.000, Akaike Information Criterion (AIC) = 1010.73, Standard Root Mean Square Residual

(SRMR) = 0.020. See Figure 2 below for standardized estimates and p-values.

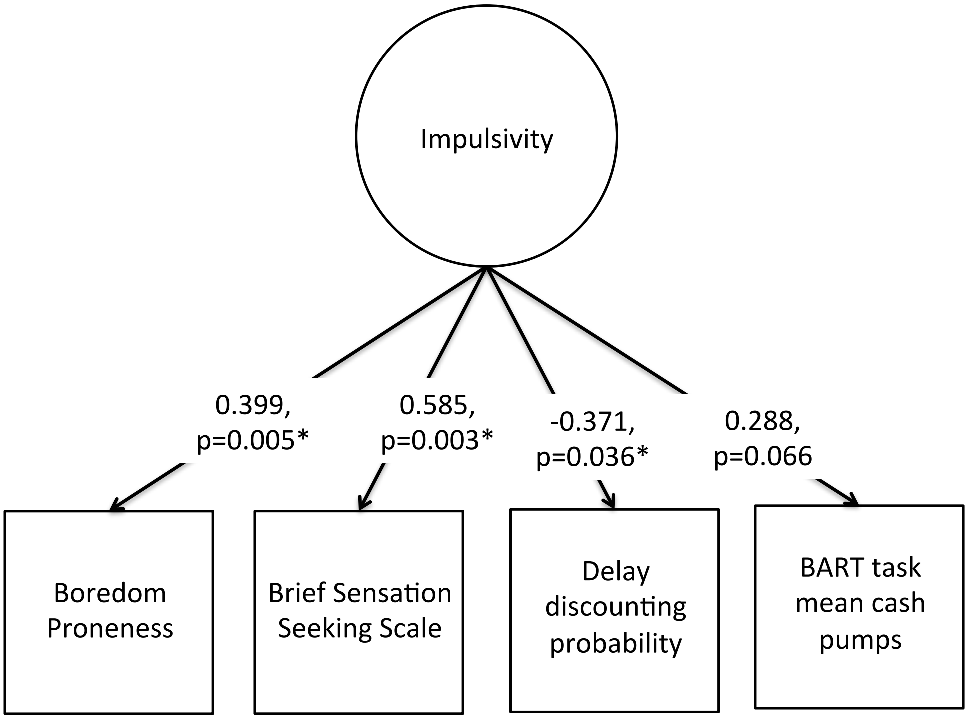

Figure 2: Graphical depiction of the latent impulsivity factor at pretest. Arrows show the standardized estimates and the p-value.

We were also successful in modelling a theory-driven latent empathy factor at pretest, consisting of the following manifest indicators: Reading the Mind in the Eyes Test, Balanced Emotional Empathy Scale, Interpersonal Reactivity Index Perspective Taking Scale and Empathic Concern Scale. However, although all manifest variables showed significant factor loadings, the commonly used indicators of model fit were not overly convincing: Root Mean Square Error of Approximation (RMSEA)=0.117, 90% confidence interval: 0.000-0.265, Comparative Fit Index (CFI) =0.959, Akaike Information Criterion (AIC) = 978.88, Standard Root Mean Square Residual (SRMR) = 0.050. See Figure 3 below for standardized estimates and p-values.

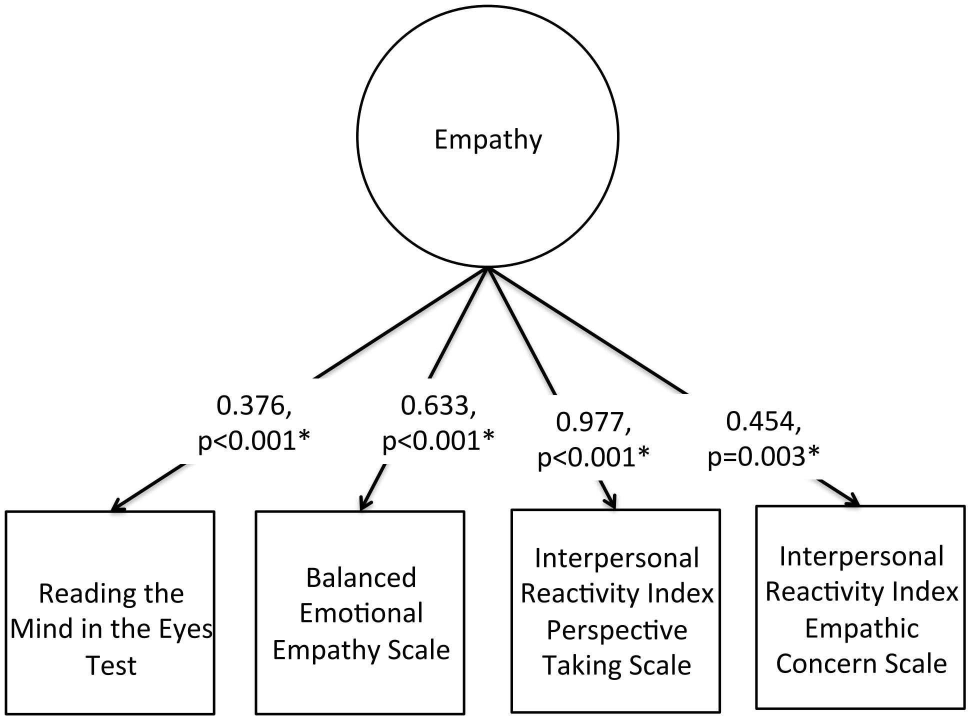

Figure 3: Graphical depiction of the latent empathy factor at pretest. Arrows show the standardized estimates and the p-value.

These results convince us of the fact that the present data set does reflect expected and reasonable relationships between questionnaires and behavioural tasks, However, both factor models were not invariant over measurement time points, which would have made our time x group interaction analysis difficult.

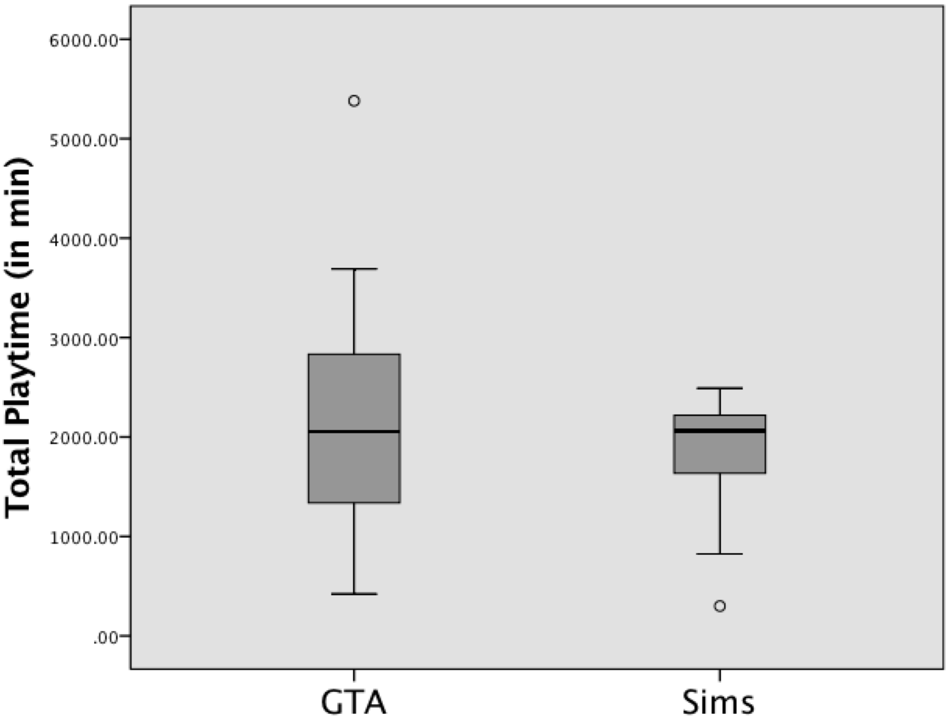

Figure 4: The black line in the middle indicates the median of the distribution, the lower part of the dark grey box represents the 25. percentile, the upper part the 75. percentile. This means that 50% of the cases are represented within the grey box. Inside the whiskers usually 95% of the cases are presented. The points are outliers (1.5 above or below the interquartile range).

References

1. Buss AH, Perry M. The Aggression Questionnaire. *J Pers Soc Psychol*, vol. 63. American Psychological Association1992, p 452.

2. Anderson CA, Deuser WE, DeNeve KM. Hot temperatures, hostile affect, hostile cognition, and arousal: Tests of a general model of affective aggression. . *Personality and Social Psychology Bulletin* 1995; **21:** 434-448.

3. Anderson CA, Carnagey NL. Causal effects of violent sports video games on aggression: Is it competitiveness or violent content? *Journal of Experimental Social Psychology*, vol. 452009, pp 731-739.

4. Anderson CA, Anderson KB, Deuser WE. Examining an Affective Aggression Framework Weapon and Temperature Effects on Aggressive Thoughts, Affect, and Attitudes. *Personality and Social Psychology Bulletin* 1996; **22**(4)**:** 336-376.

5. Payne DL, Lonsway KA, Fitzgerald LF. Rape Myth Acceptance: Exploration of Its Structure and Its Measurement Using the Illinois Rape Myth Acceptance Scale. *Journal of Research in Personality* 1999; **33**(1)**:** 27-68.

6. McMahon S, Farmer GL. An Updated Measure for Assessing Subtle Rape Myths. *Social Work Research* 2011; **35**(2).

7. Oh E, Neville H. Development and validation of the Korean Rape Myth Acceptance Scale. *The Counseling Psychologist* 2004; **32:** 301-331.

8. Detert JR, Trevino LK, Sweitzer VL. Moral disengagement in ethical decision making: a study of antecedents and outcomes. *J Appl Psychol* 2008; **93**(2)**:** 374-391.

9. Bandura A, Barbaranelli C, Caprara G, Pastorelli C. Mechanisms of moral disengagement in the exercise of moral agency. *Journal of Personality and Social Psychology* 1996; **71:** 364-374.

10. Bandura A. Social foundations of thought and action: A social cognitive theory. *Englewood Cliffs, NJ: Prentice-Hall* 1986.

11. Moore C, Detert JR, Trevino LK, Baker VL, Mayer DM. Why employees do bad things: Moral disengagement and unethical organizational behavior. *Personnel Psychology* 2012; **65:** 1-48.

12. Rosenzweig S. The picture-association method and its application in a study of reactions to frustration. *Journal of Personality* 1945; **14:** 23.

13. Hörmann H, Moog W. Der Rosenzweig P-F Test für Erwachsene deutsche Bearbeitung *Göttingen: Hogrefe* 1957.

14. Anderson CA, Dill KE. Video games and aggressive thoughts, feelings, and behavior in the laboratory and in life. *J Pers Soc Psychol*, vol. 78. American Psychological Association2000, pp 772-790.

15. Anderson CA, Carnagey NL, Flanagan M, Benjamin AJ, Eubanks J, Valentine JC. Violent video games: Specific effects of violent content on aggressive thoughts and behavior. *Advances in Experimental Social Psychology* 2004; **36:** 199-249.

16. Przybylski AK, Deci EL, Rigby CS, Ryan RM. Competence-impeding electronic games and players' aggressive feelings, thoughts, and behaviors. *J Pers Soc Psychol*, vol. 106. American Psychological Association2014, p 441.

17. Bitsakou P, Antrop I, Wiersema JR, Sonuga-Barke EJ. Probing the limits of delay intolerance: preliminary young adult data from the Delay Frustration Task (DeFT). *J Neurosci Methods* 2006; **151**(1)**:** 38-44.

18. Hoyle RH, Stephenson MT, Palmgreen P, Lorch EP, Donohew RL. Reliability and validity of a brief measure of sensation seeking. *Personality and Individual Differences* 2002; **32:** 401-414.

19. Farmer R, Sundberg ND. Boredom Proneness: The Development and Correlates of a New Scale. *Journal of Personality Assessment* 1986; **50:** 4-17.

20. Lejuez CW, Read JP, Kahler CW, Richards JB, Ramsey SE, Stuart GL *et al.* Evaluation of a behavioral measure of risk taking: the Balloon Analogue Risk Task (BART). *J Exp Psychol Appl*, vol. 82002, pp 75-84.

21. Richards JB, Zhang L, Mitchell SH, de Wit H. Delay or probability discounting in a model of impulsive behavior: effect of alcohol. *J Exp Anal Behav* 1999; **71**(2)**:** 121-143.

22. Ainslie G. Specious reward: a behavioral theory of impulsiveness and impulse control. *Psychol Bull* 1975; **82**(4)**:** 463-496.

23. Mazur JE. An adjusting procedure for studying delayed reinforcement. In: Commons ML, Mazur JE, Nevin JA, Rachim H (eds). *Quantitative analysis of behavior: Vol. 5 The effects of delay and of intervening events of reinforcement value*. Erlbaum: Hillsdale, NJ, 1987.

24. Davis MH. A multidimensional approach to individual differences in empathy. *JSAS Catalog of Selected Documents in Psychology* 1980; **10:** 85.

25. Mehrabian A. Manual for the Balanced Emotional Empathy Scale (BEES). *(Avaulable from Albert Mehrabian, 1130 Alta Mesa Road, Monterey, CA, USA 93940)* 1996.

26. Baron-Cohen S, Wheelwright S, Hill J, Raste Y, Plumb I. The "Reading the Mind in the Eyes" Test revised version: A study with normal adults, and adults with Asperger syndrome or high-functioning autism. *J Child Psychol Psychiatry* 2001; **42:** 241-251.

27. Prevost M, Carrier ME, Chowne G, Zelkowitz P, Joseph L, Gold I. The Reading the Mind in the Eyes test: validation of a French version and exploration of cultural variations in a multi-ethnic city. *Cogn Neuropsychiatry* 2014; **19**(3)**:** 189-204.

28. Buhrmester D, Furman W, Reis H, Wittenberg MT. Five domains of interpersonal competence in peer relations. *Journal of Personality and Social Psychology* 1988; **55**(6)**:** 991-1008.

29. Richardson DR, Green LR, Lago T. The relationship between perspective-taking and non-aggressive responding in the face of an attack. *Journal of Personality* 1998; **66:** 235-256.

30. Hautzinger M, Bailer M, Worall H, Keller F. Beck-Depressions-Inventar (BDI). Testhandbuch der deutschen Ausgabe. *Bern: Huber* 1995.

31. Beck AT, Steer RA, Carbin MG. Psychometric properties of the Beck Depression Inventory: Twenty-five years of evaluation. *Clinical Psychology Review* 1988; **8**(1)**:** 77-100.

32. Beck AT, Steer RA, Brown GK. Manual for the Beck Depression Inventory-II. *San Antonio, TX: Psychological Corporation* 1996.

33. Spielberger CD, Spielberger CD, Sydeman SJ, Sydeman SJ, Owen AE, Owen AE *et al.* Measuring anxiety and anger with the State-Trait Anxiety Inventory (STAI) and

the State-Trait Anger Expression Inventory (STAXI). Lawrence Erlbaum Associates Publishers1999.

34. Lorenz RC, Gleich T, Buchert R, Schlagenhauf F, Kuhn S, Gallinat J. Interactions between glutamate, dopamine, and the neuronal signature of response inhibition in the human striatum. *Hum Brain Mapp* 2015.

35. Logan GD, Van Zandt T, Verbruggen F, Wagenmakers E-J. On the ability to inhibit thought and action: general and special theories of an act of control. *Psychological Review*, vol. 911984, pp 295-327.

36. Verbruggen F, Chambers CD, Logan GD. Fictitious inhibitory differences: how skewness and slowing distort the estimation of stopping latencies. *Psychol Sci* 2013; **24**(3)**:** 352-362.

37. Bush G, Shin LM. The Multi-Source Interference Task: an fMRI task that reliably activates the cingulo-frontal-parietal cognitive/attention network. *Nat Protoc* 2006; **1**(1)**:** 308-313.

38. King JA, Colla M, Brass M, Heuser I, von Cramon D. Inefficient cognitive control in adult ADHD: evidence from trial-by-trial Stroop test and cued task switching performance. *Behav Brain Funct* 2007; **3:** 42.
